# Detrimental Effect of Sitagliptin Induced Autophagy on Multiterritory Perforator Flap Survival

**DOI:** 10.3389/fphar.2020.00951

**Published:** 2020-06-26

**Authors:** Zhengtai Chen, Chenxi Zhang, Haiwei Ma, Zihuai Huang, Jiafeng Li, Junshen Lou, Baolong Li, Qi Tu, Weiyang Gao

**Affiliations:** ^1^ Department of Orthopaedics, The Second Affiliated Hospital and Yuying Children's Hospital of Wenzhou Medical University, Wenzhou, China; ^2^ Department of Orthopaedics, Zhejiang Provincial Key Laboratory of Orthpaedics, Wenzhou, China; ^3^ Department of Second Clinical Medical, The Second Clinical Medical College of Wenzhou Medical University, Wenzhou, China; ^4^ Department of Neurosurgery, The First Affiliated Hospital of Wenzhou Medical University, Wenzhou, China; ^5^ Department of First Clinical Medical, The First Clinical Medical College of Wenzhou Medical University, Wenzhou, China

**Keywords:** multiterritory perforator flap, sitagliptin, autophagy, angiogenesis, apoptosis, oxidative stress, PI3K/Akt signaling pathway

## Abstract

Multiterritory perforator flap survival is commonly applied in surgical tissue reconstructions and covering of large skin defects. However, multiple risk factors such as ischemia, reperfusion injury, and apoptosis after reconstructive surgeries cause necrosis in distal parts with outcomes ranging from poor aesthetic appearance to reconstructive failure. A few studies have reported that sitagliptin (Sit) promotes angiogenesis and inhibits apoptosis. However, little is known about Sit-induced autophagy especially on the flap model. Therefore, our study investigated the effect of Sit and its induced autophagy on the perforator flap survival. Ninety male Sprague-Dawley rats were randomly separated into control, Sit, and Sit+3-methyladenine group. Results revealed that Sit significantly promoted flap survival by enhancing angiogenesis, reducing oxidative stress, and attenuating apoptosis. In addition, flap survival was further improved after co-administration with 3-methyladenine to inhibit autophagy. Overall, our results established that Sit has positive effects in promoting survival of multiterritory perforator flap. Sit-induced autophagy was detrimental for flap survival and its inhibition may further improve flap survival.

## Introduction

Multiterritory perforator flap have been widely used in clinics for a long time to repair skin defects such as congenital disorders, trauma, tumor ablation, and diabetic foot ulcer (Cheng et al., 2017). Previous studies have revealed that necrosis usually occurs away from the boundary between potential and dynamic territory (Taylor et al., 1992; Fichter et al., 2016). Occurrence of necrosis increases the number of surgeries and is directly lead to a patient's dissatisfaction with the treatment (Gill et al., 2004; Qing et al., 2017). Choke vessel as a kind of resistant vessel that connected two contiguous angiosomes (Taylor and Palmer, 1987). Blood supply from the pedicle is consumed after crossing the first choke zone, which results in the failure of blood flow to continue to cross the second choke zone, leading to ischemia in the potential territory in early stage (Wang et al., 2017b). Then angiogenesis and choke vessel's dilation in the second choke zone causing the reperfusion injury in the potential territory. Ischemia-reperfusion (I/R) brings a burst of reactive oxygen species (ROS) aggregated and cell apoptosis increased accordingly (Suzuki et al., 2008; Sies et al., 2017). Multiple approaches have been applied by researchers trying to overcome these problems such as use of hyperbaric oxygen (Li et al., 2009; Xie et al., 2015) and delayed flap technique (Byun et al., 2020). However, little progress has been made over the past few years, and the problem has not been satisfactorily resolved. Consequently, it's necessary to find an effective way to enhance multiterritory perforator flap survival.

Sitagliptin (Sit), an oral glucose reducing agent is widely used to treat diabetes (Scott et al., 2018). However, recent research reveals that Sit not only lowers blood sugar, but it also has beneficial effects in other organs and tissues. For example, Sit promotes angiogenesis and increases blood flow in the rat critical limb ischemia model (Chua et al., 2013). Sit protects the heart from ischemia reperfusion (IR) injury in the rat cardiovascular disease model by alleviating apoptosis and oxidative stress (Chua et al., 2014) and protects the aorta by regulating AMPK and MAPK pathways to reduce inflammatory reactions (Zeng et al., 2014). Sit also has beneficial effects of inhibiting apoptosis under hypoxic conditions in a cell model of human umbilical vein endothelial cells (Nagamine et al., 2017). In addition, Sit can ameliorate hepatic insulin resistance (Zheng et al., 2018) and induce preservation of angiogenic function in endothelial progenitor cells by activating autophagy (Dai et al., 2018). Effects of Sit and Sit-induced autophagy after reconstructive flap surgery are however unknown.

Autophagy is a highly conserved process of eukaryotic cell degradation that is crucial for survival of cells under nutrient-deprived conditions (Du et al., 2012; Lahiri et al., 2019) and has increasingly attracted the attention of researchers. However, autophagy is a double-edged sword. Autophagy activation can have detrimental effects during reperfusion contrary to alleviating apoptosis and oxidative stress during ischemic period (Takagi et al., 2007). Previous studies have confirmed that autophagy performs different functions at different times. Therefore, the present study seeks to investigate the effect of Sit and Sit-induced autophagy on the survival of multiterritory perforator flap.

## Materials And Methods

### Reagents

Sit (C₁₆H₁₅F₆N₅O; purity ≥ 98%; **Figure S1**) was acquired from Yeyuan Biotech (Shanghai, China). 3-methyladenine (3MA) (C_6_H_7_N_5_; purity ≥ 98%) were purchased from Sigma-Aldrich Chemical Company (Milwaukee, WI, United States).

### Animals Details

Ninety 8-week-old healthy male Sprague Dawley rats (250–300 g) were obtained from Wenzhou Medical University (license no. SCXK[ZJ]2015-0001). Protocol used for animal experiments was in accordance with ethical guidelines on animal experimentation of Laboratory Animals of China National Institutes of Health. The rats used in this study was approved by the Animal Research Committee of Wenzhou Medical University (wydw2017–0022). All rats were housed in a clean room with a 12-h light/dark cycle at 25°C, and given food and water.

### Flap Animal Model

**Figure 1:**
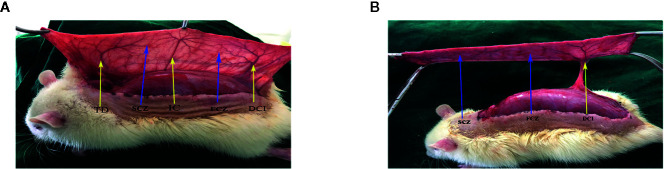
Flap model. **(A)** Three vascular territories and two choke vessel zones of the flap observed from the pedicle to distal, containing the deep circumflex iliac (DCI) territory, posterior intercostal (IC) territory, thoracodorsal (TD) territory, first choke zone (FCZ), and second choke zone (SCZ). **(B)** Only the DCI artery was retained, IC artery, and TD artery were ligated.

Rats were anesthetized through intraperitoneal injection with 3% pentobarbital sodium (60 mg/kg) and an additional dose was given when necessary during the procedure. Dorsal fur was subsequently removed by an electric shaver and depilatory agent applied before surgery. A flap pedicled on a deep circumflex iliac artery (DCIA-flap) was performed on the right side of each rat as reported previously (Tao et al., 2016). Flap model consisted of three vascular territories; (i) anatomic territory namely the deep circumflex iliac (DCI) angiosome, (ii) dynamic territory namely intercostal (IC) angiosome, and (iii) potential territory namely thoracodorsal (TD) angiosome (**Figure 1A**). Flap size was approximately 2.5 × 11 cm, and was slightly separated from the underlying fascia. TD and IC were subsequently ligated (**Figure 1B**), the flap was placed again on its donor bed and sutured to its margins with 4-0 silk. On day 7, all rats were euthanized with an overdose of pentobarbital sodium.

### Drug Administration

Ninety rats were randomly separated into control group (n=30), Sit group (n=30), and Sit+3MA group (n=30). The rats in Sit and Sit+3MA group was treated with Sit orally at a dosage of 10 mg/kg/d (dissolved in 1 ml saline) for total 7 d, while the control group with equal volume of saline using the same protocol. In order to reach the peak of plasma concentration in the initial postoperative hours the first administration of Sit was 2 h before the operation in the Sit+3MA and Sit group (Beconi et al., 2007). Rats in the Sit+3MA group received 3MA at a dosage of 10 mg/kg/d (dissolved in 1 ml saline) 30 min before Sit administration (Wang et al., 2017a) through intraperitoneal injection while the control and Sit group with equal volume of saline using the same protocol.

### Flap Survival Evaluation

Rats were anesthetized and high-quality photographs of the dorsal skin flap obtained using a digital camera on the 3^rd^ and 7^th^ postoperative days (PODs). Survival and ischemic areas were evaluated using Image-Pro Plus imaging software (ver. 6.0; Media Cybemetics), and the percentage survival area calculated as × 100%.

### Tissue Edema Measurement

Tissue edema was assessed by measuring the water content of the flap. Flap specimens (n=6) were harvested and dehydrated in an autoclave at 50 °C on the 7^th^ POD. Samples were weighed daily until a constant weight was reached over 2 d. Water content was calculated as [(wet weight−final dry weight) ÷ initial weight] × 100%.

### Hematoxylin and Eosin (H&E) Staining

Rats were euthanized with overdose pentobarbital sodium (Solarbio Science & Technology, Beijing, China) after 7 d, six tissue samples excised from the middle part of the second choke vessel zone (SCV) in each group (0.5 cm × 0.5 cm) and post-fixed in 4% (v/v) paraformaldehyde for 24 h, and embedded in paraffin wax. Samples were then cut into tissue sections of 4 µm thickness and stained with H&E using standard histology protocols (Solarbio Science & Technology, Beijing, China). Six randomly selected fields from three random sections of angiogenesis were observed under a light microscope (Olympus Corp, Tokyo, Japan). In addition, number of microvessels per unit area (/mm^2^) was calculated as a measure of microvascular density (MVD).

### Immunohistochemistry (IHC)

Six samples from SCV in each group were deparaffinized in xylene and rehydrated by a series of ethanol concentrations from 100% to 75%. Sections were subsequently immersed in 3% hydrogen peroxide solution to inhibit endogenous peroxidase activities and incubated in 10% normal goat serum. Finally, sections were incubated at 4°C overnight against the following primary antibodies: anti-Cadherin 5 (1:100, Boster Biological Technology, China), anti-CD34 (1:200, Abcam, UK), anti-VEGF (1:200, Abcam), anti-CTSD (1:200, Proteintech, USA), anti-SOD1 (1:200, Proteintech), and anti-C-CASP3 [1:200, CST (Cell Signaling Technologies, USA)]. These sections were then incubated with HRP-conjugated secondary antibody (Santa Cruz Biotechnology, Dallas, USA) and counterstained with hematoxylin. Each specimen was imaged at a magnification of ×200 using a DP2-BSW image-acquisition system (Olympus Corp), and absorption values quantified using the Image-Pro Plus software (Media Cybernetics, MD, USA) to assess expressions of Cadherin 5, VEGF, CTSD, SOD1, and C-CASP3 and the number of CD34 positive microvessels.

### Immunofluorescence

Six specimens were deparaffinized and rehydrated as described in the procedure for IHC. A solution of 3% (v/v) hydrogen peroxide was applied and tissue antigen retrieved with 10.2 mM sodium citrate buffer. This was followed by application of 0.1% (v/v) PBS-Triton X-100 to permeate samples. Slides were incubated at 4°C overnight with a primary antibody against LC3II (1:200, Sigma, Milwaukee, USA) and α-SMA (1:200, Proteintech, USA) after blocking in 10% (v/v) normal goat serum for 1 h. Specimens were subsequently incubated with a secondary antibody at room temperature for 1 h and nuclei stained with DAPI (Beyotime Biotechnology, Jiangsu, China). All images were evaluated under a fluorescence microscope (Olympus, Tokyo, Japan). Six random fields of three random sections from each tissue sample were calculated as a percentage of LC3II-positive cells and α-SMA positive microvessels.

### Laser Doppler Perfusion Image

Laser Doppler blood flow (LDBF) measurements were performed to assess blood flow in the flap. A laser Doppler imager (Moor Instruments, Axminster, UK) was used to scan a full field of the flap. Rats were placed in a prone position under anesthesia. Blood flow images on the 0, 3^rd^, and 7^th^ PODs were processed to provide a color-coded living flux image, and blood perfusion at SCV measured. Each rat was scanned three times and the mean value calculated.

### Flap Angiography

On the 7^th^ POD, rats (n=6) underwent whole-body angiography. Lead oxide-gelatin (80 ml/kg) was perfused into the common carotid artery *via* a silicone rubber catheter until the limbs turned yellow. Fixed at 4°C overnight, then the flap was harvested and radiographed using an X-ray machine (54 kVp, 40 mA, 100 s exposure). The first choke zone (FCZ) and second choke zone (SCZ) were divided into four 0.5×0.5 cm squares. The number of choke vessel that crossed each standardized square was counted to determine choke vessel density.

### Measurement of Superoxide Dismutase (SOD), Glutathione (GSH), and Malondialdehyde (MDA) level

SOD, GSH, and MDA test kits purchased from Nanjing Jiancheng Biology Jiancheng Technology Institution (Nanjing, China) were used to assess the oxidative stress of flap. Six samples from SCV were weighed, homogenized, and centrifuged at 3,500 rpm for 15 min dilution with saline on the 7^th^ POD. SOD activity, GSH level, and MDA content were subsequently determined as previously described (Tao et al., 2016).

### Western Blotting

Tissue samples (n = 6) from rats in the Sit, Sit+3MA, and control groups were harvested from the middle of SCV for western blotting. Samples were homogenized in RIPA buffer containing 1 mM phenylmethanesulfonyl fluoride and the concentrations of the protein were detected by BCA protein assay kit (Thermo Fisher Scientific, Rockford, IL, USA). Proteins were separated by 12% (w/v) gel and then transferred onto PVDF membranes (Bio-Rad, USA). This was followed by blocking in 5% skimmed milk for 120 min at room temperature and overnight incubation at 4°C with the following primary antibodies: VEGF (1:1,000), Cadherin 5 (1:1,000), LC3II (1:500), SOD1 (1:1,000), C-CASP3 (1:1,000), MMP9 (1:1,000, Abcam, UK), SQSTM1/p62 (1:1,000, Abcam), Bax (1:1,000, Proteintech, USA), CTSD (1:1,000, Proteintech), HO1 (1:1,000, Proteintech), Bcl-2 (1:1000, CST), eNOS (1:1,000, CST), VPS34 (1:1,000, CST), Beclin1 (1:1,000, CST), PI3K (1:1,000, CST), p-PI3K (1:1,000, CST), AKT (1:1,000, CST), p-Akt (1:1,000, CST), mTOR (1:1,000, CST), p-mTOR (1:1,000, CST), and GAPDH (1:1,000, Biogot Technology, China) as a loading control. Membranes were subsequently incubated with secondary antibody for 2 h at room temperature. Bands was visualized using ECL reagent kit (PerkinElmer Life Sciences, Waltham, USA). Finally, the intensity of blots was analyzed using Image Lab 3.0 software (Bio-Rad, Hercules, USA).

### Statistical Analysis

Statistical analysis was conducted using SPSS statistical software package (version 22.0; Chicago, IL, United States). All data are presented as means ± standard error. Comparison of means between two groups was performed using independent-sample *t*-test. *P*-values less than 0.05 were considered statistically significant.

## Results

### Sit and 3MA Ameliorate Perforator Flap Survival

**Figure 2:**
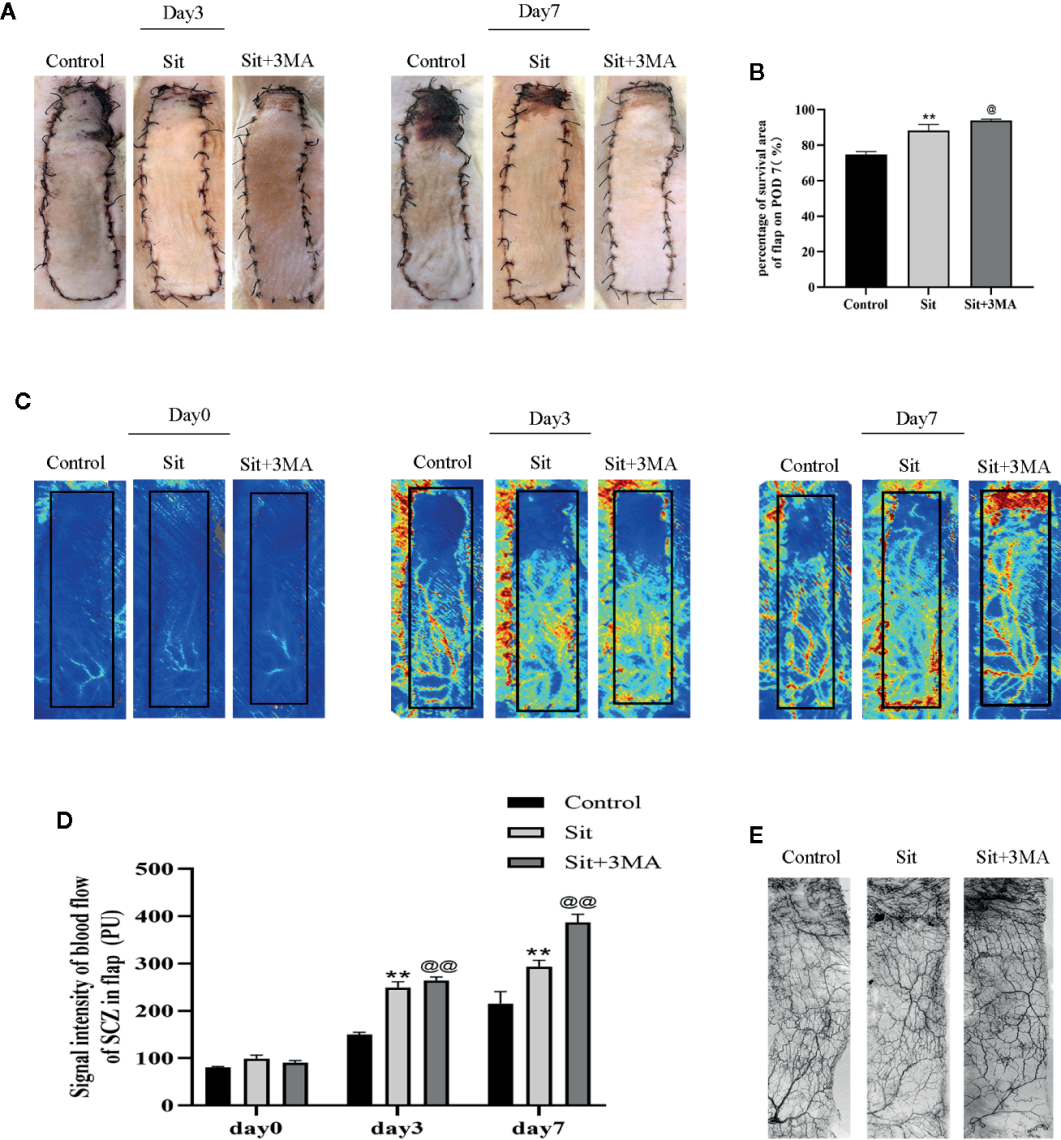
Sit and 3MA ameliorate multiterritory perforator flap survival. **(A)** Digital photographs of flap survival/necrosis area on the 3^rd^ and 7^th^ PODs in the control, Sit, and Sit+3MA groups. **(B)** The percentages of survival area in each group. **(C)** LDBF imaging showing vascular flow and blood supply in a perforator flap on the 0, 3^rd^, and 7^th^ PODs in the control, Sit, and Sit+3MA groups. **(D)** Histogram representing the quantified signal intensity on the 0, 3^rd^, and 7^th^ PODs. **(E)** Flap angiograms on the 7^th^ PODs and the vasculature was in a mass in the necrosis area. **p < 0.01, vs control group; ^@^p < 0.05 and ^@@^ < 0.01, vs Sit group. Data are expressed as mean ± standard error, n = 6 per group.

All rats survived after flap surgery without developing any postoperative infection. Necrosis was not evident in the skin flaps of rats from all groups but the distal part of flaps became swollen and pale on the 3^rd^ POD (**Figure 2A**). Tissue necrosis appeared dry, dark, crumpled, and the boundary between survival and necrosis gradually expanded from the top of the flap to the pedicle and stabilized on the 7^th^ POD (**Figure 2A**). Survival rate was higher in the Sit group than in the control group and was greatly enhanced in Sit+3MA group (**Figure 2B**). Laser Doppler results exhibited microvascular network reconstruction and blood flow (**Figure 2C**) and the data of measurements directly after the operation showed no differs significantly among three groups. However, on 3^rd^ and 7^th^ PODs blood flow was higher in the Sit+3MA group than in the Sit and control groups, and was lowest in the control group (**Figure 2D**). Additionally, the vasculature showed the best filling with contrast agent in the Sit +3MA group while in the other two groups the vasculature in the potential territory was mass in part or whole (**Figure 2E**). By contrast, tissue water content on the 7^th^ POD was significantly higher in the control group than in the Sit group, with the lowest tissue water content recorded in the Sit+3MA group (**Figures S2A, B**). Overall, our results indicated that Sit treatment reduces tissue edema, increases blood flow and promotes flap survival, and these effects are further enhanced through co-administration with 3MA.

### Sit Induces Autophagy in the Perforator Flap

**Figure 3:**
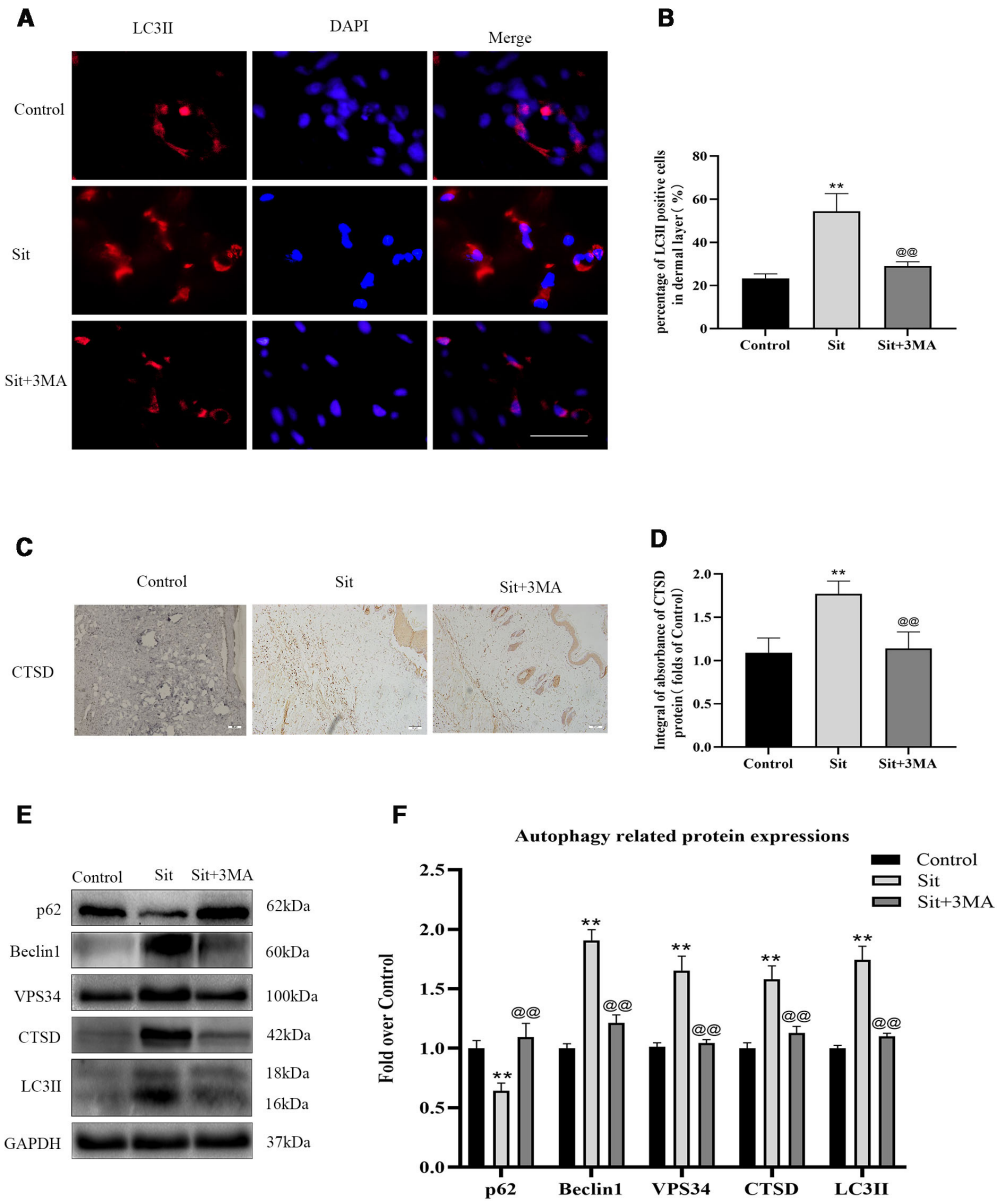
Sit induces autophagy in the perforator flap. **(A)** Autophagosomes (red) in cells in SCZ of flaps in the control, Sit, and Sit+3MA groups by immunofluorescence staining for LC3II (scale bar: 20 µm). **(B)** Histogram of fluorescence intensity of LC3II-positive cells in the dermal layer in each group. **(C)** IHC staining of CTSD expression in the dermis (original magnification: 200×; scale bar: 50 µm). **(D)** Histogram of CTSD level estimated by IHC. **(E)** Western blotting for Beclin1, VPS34, CTSD, SQSTM1/p62, and LC3II expressions in flap of the control, Sit, and Sit+3MA groups. All gels have been run under the same electrophoretic conditions and cropped blots are used here. **(F)** Histogram of autophagy related protein expressions of Beclin1, p62, CTSD, VPS34, and LC3II calculated as the folds of control. **p < 0.01, vs control group; ^@@^p < 0.01, vs Sit group. Data are presented as mean ± standard error, n = 6 per group.

Autophagy related proteins were analyzed to investigate activation of autophagy in the perforator flap. In this study, we analyzed essential components of autophagosomes against Beclin1, VPS34, and LC3II protein levels, and autophagic substrate protein SQSTM1/p62 to detect autophagy flow and autolysosome related protein CTSD. Immunofluorescence was used to detect the frequency of LC3II positive cells in the dermis (**Figure 3A**). Our results indicated that more autophagosomes were activated in the Sit group than in the control group (**Figure 3B**). In addition, IHC findings revealed that integral absorbance of CTSD was higher in the Sit group than in the control group (**Figures 3C, D**). Western blotting exhibited a lower level of p62, but higher levels of Beclin1, VPS34, CTSD, and LC3II in the Sit group than in the control group (**Figures 3E, F**). However, Sit+3MA group displayed same results as the control group. Immunofluorescence results indicated that the frequency of LC3II positive puncta in the dermis was lower in the Sit+3MA group than in Sit group (**Figures 3A, B**). In addition, both IHC and western blotting results revealed that 3MA treatment decreased CTSD levels (**Figures 3C–F**). Western blotting was further performed to investigate the expressions of p62, Beclin1, VPS34, and LC3II and to verify if 3MA successfully inhibited Sit-induced autophagy (**Figure 3E**). Results revealed higher levels of p62 expression but lower levels of Beclin1, LC3II, and VPS34 in the flap tissues after co-administration with 3MA (**Figure 3F**). Our findings collectively prove that Sit can induce autophagy and 3MA effectively inhibits Sit-induced autophagy.

### Effect of Sit-Induced Autophagy on Angiogenesis in the Perforator Flap

**Figure 4:**
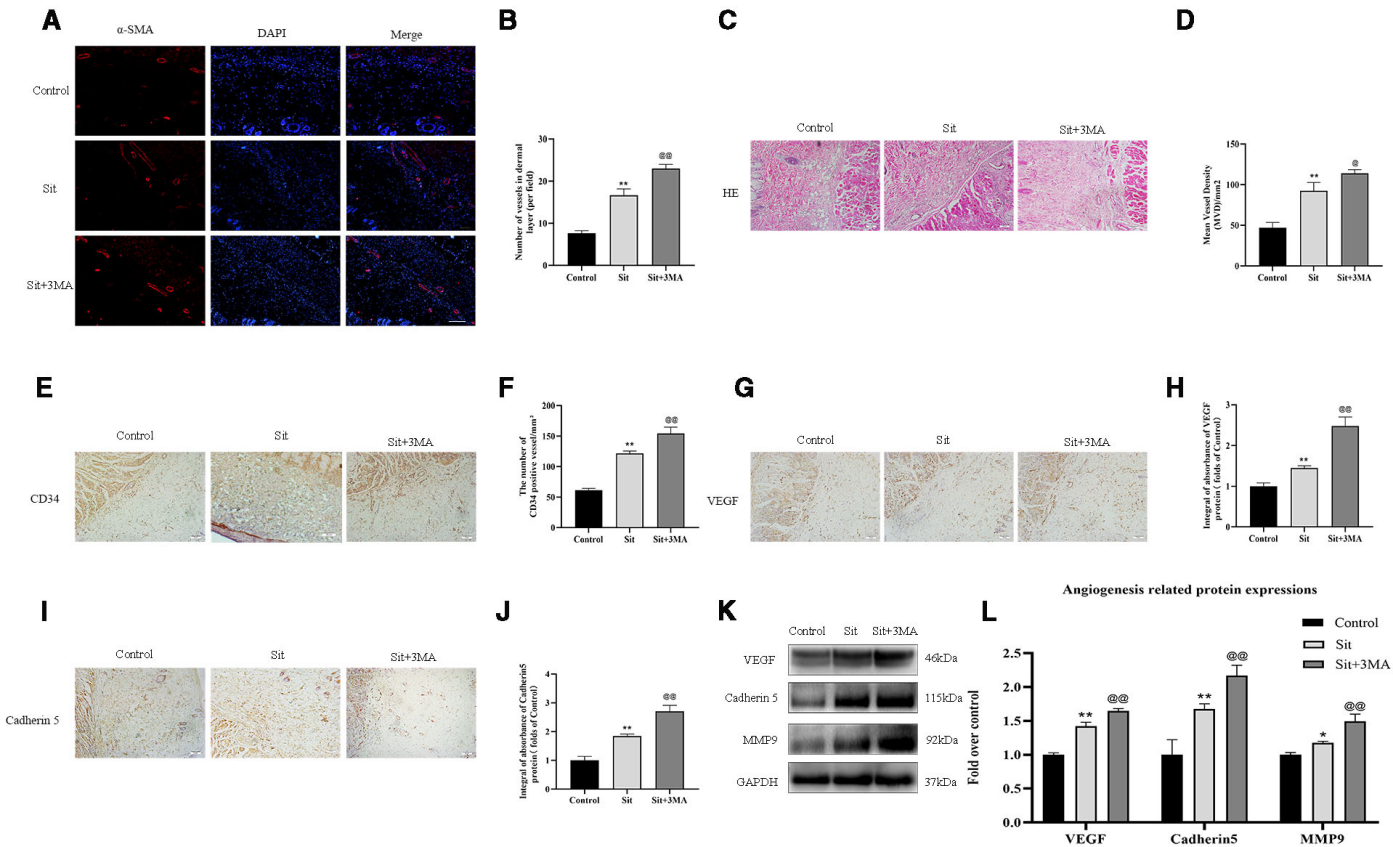
Effect of Sit-induced autophagy on angiogenesis in the perforator flap. **(A)** Microvessels (red) in SCZ of flaps in the control, Sit, and Sit+3MA groups were estimated by immunofluorescence staining for α-SMA in the dermal layer (scale bar: 20 µm). **(B)** Histograms representing percentages of α-SMA labeled microvessels in each group. **(C)** H&E staining exhibiting subcutaneous histology of the flap, showing microvessels in SCZ in the control, Sit, and Sit+3MA groups (original magnification ×200; scan bar, 50 μm). **(D)** Histogram indicating percentage of mean vessel density in each group. **(E)** IHC for CD34 positive vessels in the control, Sit, and Sit+3MA groups (original magnification ×200; scale bar, 50 µm). **(F)** Histogram of the percentage of CD34-positive vessel density in each group. **(G, I)** IHC for VEGF and Cadherin 5 expression in the flap in the control, Sit, and Sit+3MA groups (original magnification ×200; scale bar, 50 µm). **(H, J)** The optical density values of VEGF and Cadherin 5 in each group. **(K)** The expressions of MMP9, VEGF, and Cadherin 5 detected by western blotting in the control, Sit, and Sit+3MA groups. All gels have been run under the same experimental conditions and cropped blots are used here. **(L)** Histogram of the optical density values of MMP9, VEGF, and Cadherin 5 in each group. *p < 0.05 and **p < 0.01, vs control group; ^@^p < 0.05 and ^@@^p < 0.01, vs Sit group. Data are presented as mean ± standard error, n = 6 per group.

We measured levels of various angiogenesis markers to detect the mechanism responsible for the effect of Sit-induced autophagy on angiogenesis. Immunofluorescence was performed to determine α-SMA positive microvessels (**Figure 4A**). The quantity of α-SMA positive microvessels was higher in the Sit and Sit+3MA groups than in the control group and Sit+3MA group recorded the highest levels of α-SMA positive microvessels (**Figure 4B**). This phenomenon was also verified through H&E and IHC staining of CD34-positive vessels (**Figures 4C–F**). Results from IHC and western blotting experiments indicated that VEGF and Cadherin 5 expressions were higher in the Sit group than in the control group, with highest expression levels observed in the Sit+3MA group (**Figures 4G–L**). In addition, optical density value of MMP9 was higher in the Sit group than in the control group, and highest value were recorded in Sit+3MA group (**Figures 4J, L**). Overall, our results illustrated that Sit promotes angiogenesis and inhibits Sit-induced autophagy, and that the pro-angiogenic effects can be enhanced further in the perforator flap.

### Effect of Sit-Induced Autophagy on Oxidative Stress in the Perforator Flap

**Figure 5:**
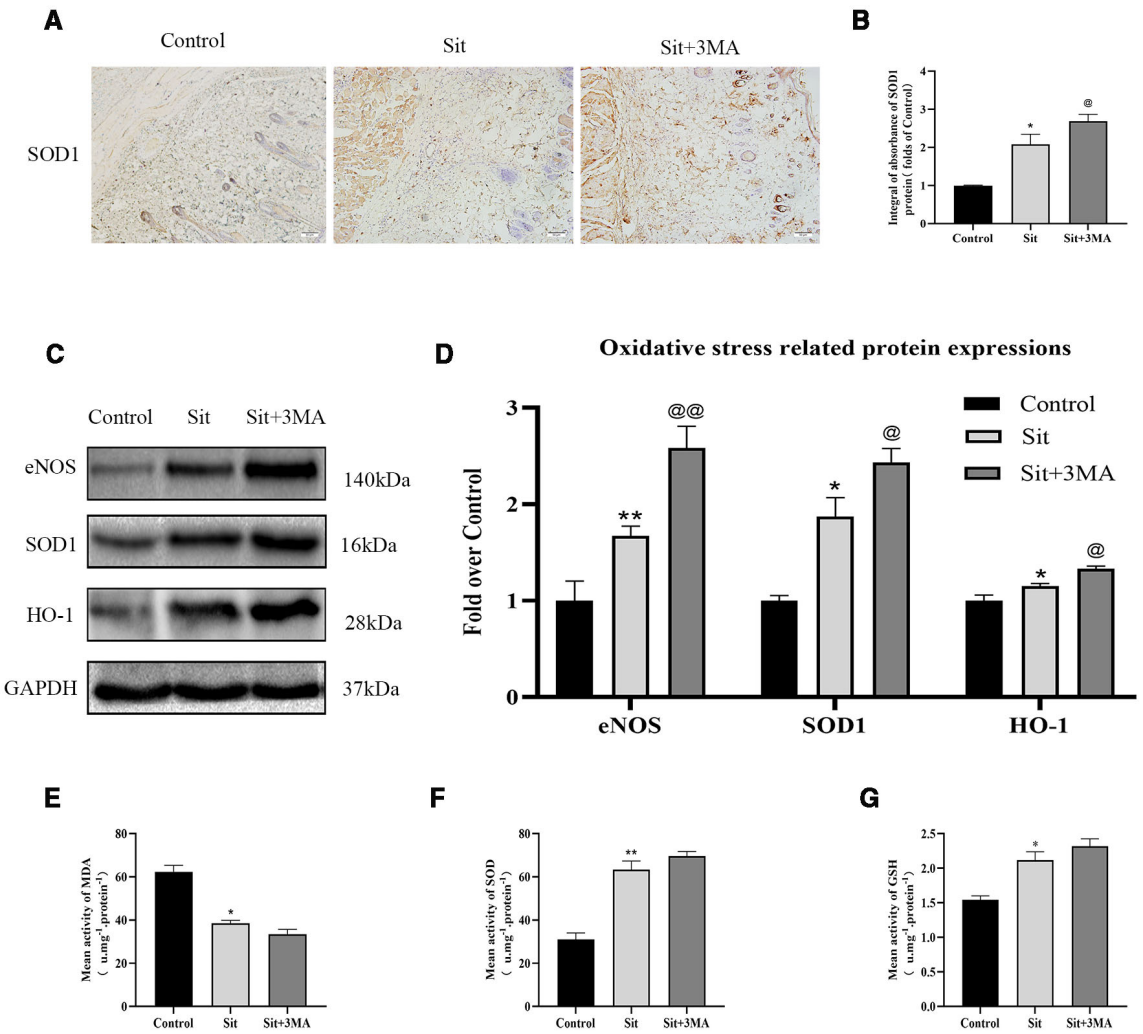
Effect of Sit-induced autophagy on oxidative stress in the perforator flap. **(A)** IHC for SOD1 expression in the perforator flap in the control, Sit, and Sit+3MA groups (original magnification ×200; scale bar, 50 µm). **(B)** Histogram of optical density values of SOD1 quantified and analyzed by IHC. **(C)** The expressions of SOD1, eNOS, and HO1 as revealed by western blotting in the control, Sit, and Sit+3MA groups. Gels have been run under the same experimental conditions and cropped blots are used here. **(D)** Histogram of oxidative stress related protein expressions of SOD1, eNOS, and HO1 in each group. **(E)** MDA level evaluated using modified thiobarbituric acid test. **(F)** Total SOD activity evaluated using xanthine oxidase method. **(G)** GSH level evaluated using modified 5,5'-dithiobis method. *p < 0.05 and **p < 0.01, vs control group; ^@^p < 0.05 and ^@@^p < 0.01, vs Sit group. Data are presented as mean ± standard error, n = 6 per group.

IHC and western blotting were performed for SOD1 expression to evaluate oxidative stress level. Results indicated a higher level of SOD1 expression in the Sit group than in the control group. And level of SOD1 was considerably higher in the Sit+3MA group than in the Sit group (**Figures 5A–D**). We subsequently evaluated expression levels of eNOS and HO1 in the flap. Protein levels of eNOS and HO1 were higher in the Sit group than in the control group, with highest levels recorded in the Sit+3MA group (**Figures 5C, D**). In addition, increased ROS consumed two endogenous antioxidative chemicals, SOD and GSH, augmenting the production of MDA. We consequently tested SOD, GSH, and MDA contents using corresponding kits to estimate oxidative stress levels. Results demonstrated that average levels of SOD and GSH content were higher and MDA level was lower in the Sit group (**Figures 5F, G**) than in the control group (**Figure 5E**). At the same time, levels of SOD and GSH were also higher in Sit+3MA group with lower levels of MDA than in Sit group, but difference was not significant (**Figures 5E–G**). Overall, Sit significantly reduces oxidative stress, and inhibition of Sit-induced autophagy further enhances the anti-oxidative stress effects of Sit in the perforator flap.

### Effect of Sit-Induced Autophagy on Apoptosis in the Perforator Flap

**Figure 6:**
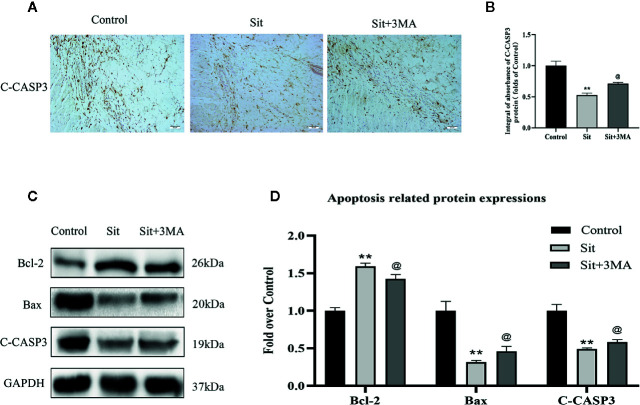
Effect of Sit-induced autophagy on apoptosis in the perforator flap. **(A)** Expression of C-CASP3 in the flap assessed by IHC in the control, Sit, and Sit+3MA groups (original magnification ×200; scale bar, 50 µm). **(B)** Histogram of integral absorbance of C-CASP3 protein in each group. **(C)** Application of western blotting to evaluate the expressions of apoptosis-related proteins of Bax, Bcl-2, and C-CASP3. Gels have been run under the same experimental conditions and cropped blots are used here. **(D)** Quantification of optical density values of Bax, Bcl-2, and C-CASP3 in the control, Sit, and Sit+3MA groups. **p <0.01, vs control group; ^@^p lt; 0.05 vs Sit group. Data are presented as mean ± standard error, n = 6 per group.

Immunohistochemical staining and western blotting techniques were used to investigate expression of apoptosis-related proteins. IHC revealed decreased integral absorbance of C-CASP3 in the Sit group (**Figures 6A, B**). Moreover, western blotting results indicated that levels of C-CASP3 and Bax were lower in Sit group than in the control group (**Figures 6C, D**). The level of anti-apoptotic protein, Bcl-2 was higher in the Sit group than in control group (**Figures 6C, D**). Strikingly, IHC revealed that effects of Sit on apoptosis were reversed after inhibition of Sit-induced autophagy (**Figures 6A, B**). Besides, western blotting confirmed IHC results (**Figures 6C, D**). Collectively, our results indicate that Sit can reduce apoptosis to inhibit cell-death and 3MA ablates the autophagy activating effects of Sit.

### Sit Regulates the PI3K/Akt Signaling Pathway to Attenuate Apoptosis

**Figure 7:**
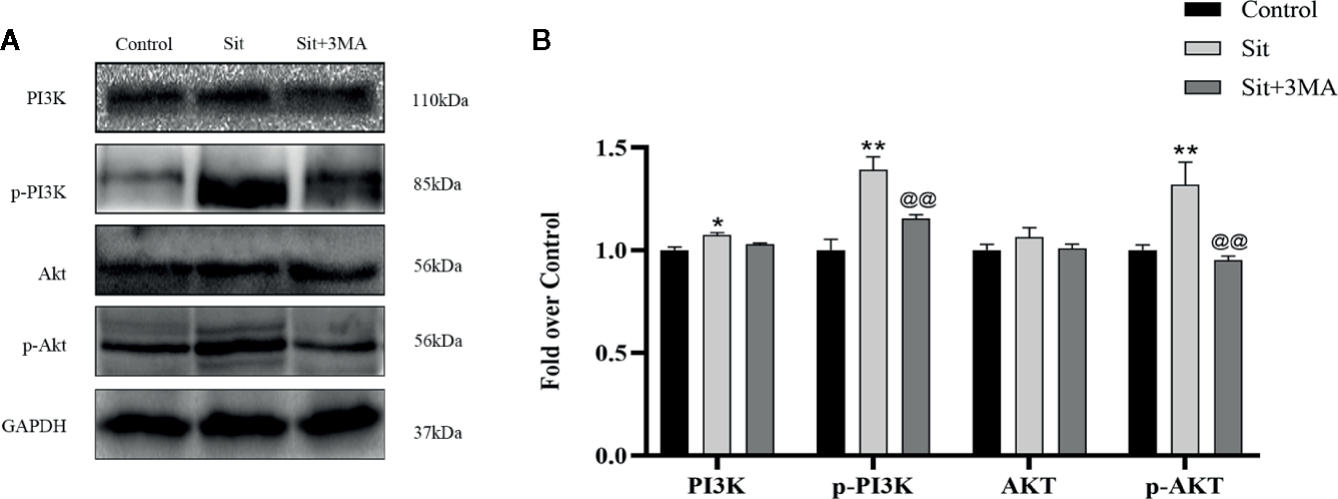
Sit regulates the PI3K/Akt signaling pathway to attenuate apoptosis. **(A)** Expressions of PI3K, p-PI3K, Akt, and p-Akt in the flap using western blotting in the control, Sit, and Sit+3MA groups. Gels have been run under the same experimental conditions and cropped blots are used here. **(B)** Histogram representing quantification and analysis of optical density values of PI3K, p-PI3K, Akt, and p-Akt. *p < 0.05 and **p < 0.01, vs control group; ^@@^p < 0.01, vs Sit group. Data are presented as mean ± standard error, n = 6 per group.

Why was anti-apoptotic effect of Sit decreased after inhibiting autophagy? According to previous studies, Sit protects cells from apoptosis by activating PI3K/Akt signaling pathway (Zhou et al., 2018). Notably, 3MA is not only an autophagy inhibitor but also a selective PI3K inhibitor. Consequently, we applied western blotting to detect levels of PI3K/Akt pathway related proteins. Our results revealed that the expression of phosphorylated PI3K and Akt was higher in Sit group than in Sit+3MA and control groups (**Figures 7A, B**). Expression levels of PI3K and Akt were not significantly different between the three groups (**Figures 7A, B**). Additionally, the difference in expression levels of PI3K and Akt between control and Sit+3MA groups was not statistically significant (**Figures 7A, B**). Our results confirmed that Sit can alleviate apoptosis by regulating PI3K/Akt pathway in the perforator flap and this explains why anti-apoptotic effects of Sit were decreased after inhibiting autophagy.

## Discussion

Sit is a commonly used oral hypoglycemic drug (Farag et al., 2013). Sit not only has a hypoglycemic effect, but also significantly contributes to the treatment of diseases involving induction of angiogenesis in diabetic rats (Khodeer et al., 2019). In renal ischemia reperfusion injury model Sit inhibited IR-induced elevation of tumor necrosis factor-α (TNF-α), nuclear factor kappa-B (NF-κB,) and caspase-3 and reduce renal injury (Youssef et al., 2015). and ameliorates acute liver injury through suppression of inflammation and apoptosis (El-Kashef and Serrya, 2019). However, there are few studies on effects of Sit-induced autophagy especially in the flap model. We established that Sit-induced autophagy is harmful to perforator flap survival but inhibited Sit-induced autophagy can enhance positive effects of Sit on perforator flap survival.

Previous study have revealed that Sit-induced autophagy has protective effects on endothelial dysfunction (Wang et al., 2018). However, autophagy has two functions; either keeping the cells survived through degrades some substances inside the cell and or directly participating in various forms of cell death (Ai et al., 2020). A few studies have confirmed that autophagy is detrimental during reperfusion and excessive autophagy destroys essential components resulting in premature cell death (Matsui et al., 2007; Mankarious et al., 2016). Therefore, several autophagy related indicators are evaluated to determine levels of autophagy including Beclin 1, LC3II, and VPS34 during the sequestration process (Chun and Kim, 2018), p62 is a marker for autophagic transportation and degradation process (Lamark et al., 2017) and CTSD is a marker for autolysosomes (Kanamori et al., 2011). Our results indicated that expressions of Beclin 1, LC3II, VPS34, and CTSD proteins in the Sit group were higher compared to the control group. However, Sit+3MA group displayed contrasting results implying that Sit can promote autophagy and 3MA can effectively inhibit Sit-induced autophagy.

A rapid vascularization to restore blood perfusion is significant to avoid flap necrosis (Zhou et al., 2019). Consequently, it's essential to evaluate Sit's pro-angiogenesis ability, and the relationship between angiogenesis and Sit-induced autophagy in a perforator flap. Angiogenesis involves several cellular processes, and MMP9 promotes dissociation of cell junctions by degrading the extracellular matrix and facilitates VEGF release to some extent (HP et al., 2019). VEGF serves as an initiating signal that induces mitosis in endothelial cells, and increases microvascular permeability (Shi et al., 2016; Stacker et al., 2016). Cadherin 5 forms intercellular junctions (Orsenigo et al., 2012). Our results displayed substantial increases in MMP9, VEGF, and Cadherin 5 protein levels after treatment with Sit, and protein levels were further increased after co-administration with 3MA. However not only angiogenesis, choke vessel's remodeled and inosculation also played an important role in promoting multiterritory perforator flap survival (Luo et al., 2019). The expansion of choke vessel is a gradual vascular remodeling process called arteriogenesis and finally developed into true anastomoses without resistance (Simons, 2005). Our angiography result showed that Sit contributes to the choke vessel's dilation and the vasculature showed a better filling with contrast agent in the Sit and Sit+3MA groups than control group. Overall, we concluded that Sit promotes angiogenesis and arteriogenesis in the perforator flap, and inhibited Sit-induced autophagy can strengthen these effects on flap.

Unlike skin graft suffering from hypoxia only, flap transfer is subjected to both ischemia and reperfusion injury showed the aggregation of ROS, inflammation, apoptosis (van den Heuvel et al., 2009; Wang et al., 2011). In our previous study sodium fluorescein staining was delayed in the flap at early postoperation stages, especially in the SCZ (Wang et al., 2017b). This delay phenomenon eventually disappeared at 1^st^ POD in the SCZ which means that IR injury did happen in the potential territory. IR injury is a leading cause of necrosis after reconstructive flap surgery (Guo et al., 2018). Apoptosis and oxidative stress are principal causes of cell death after reperfusion (Burns et al., 1998; Tanaka et al., 2009). Autophagy is beneficial in preventing apoptosis and oxidative stress whereas insufficient or excessive autophagy can exacerbate cell death. This study has attempted to reveal the effects of Sit-induced autophagy on oxidative stress and apoptosis in the perforator flap. Firstly, present study demonstrates that Sit treatment suppresses expression of anti-oxidative stress related proteins SOD1, eNOS, and HO, and increases the level of MDA. Antioxidant effects were stronger in the Sit+3MA group than in the Sit group. These findings imply that Sit has antioxidant properties and inhibited Sit-induced autophagy can enhance Sit's effect. Secondly, western blotting and IHC results of expressions of apoptosis related proteins Bax, Bcl-2, and C-CASP3 demonstrated that Sit promotes anti-apoptosis. However, co-administration with 3MA reduces the effects of Sit on anti-apoptosis. Remarkably, antiapoptotic effect decreased after suppressing autophagy, but the survival rate of the flap was enhanced further.

Interaction between apoptosis and Sit-induced autophagy mentioned previously prompted our investigation of the mechanism by which antiapoptotic effects of Sit weaken after co-administration with 3MA. 3MA is a selective PI3K inhibitor. In addition, the PI3K/Akt signaling pathway regulates a variety of physiological processes (Ye et al., 2019). Activation of PI3K/Akt signaling pathway can inhibit caspase activation during apoptosis, and up-regulate activity of the Bcl-2 family proteins (New et al., 2007). Moreover, Sit can activate PI3K/Akt signaling pathway to protects cells from apoptosis which has been confirmed in diabetic fatty rats model (Chang et al., 2013). In the present study, we applied western blotting to detect PI3K/Akt pathway related proteins. Subsequently, our findings revealed that Sit treatment can reduce apoptosis in the perforator flap through activation of PI3K/Akt signaling pathway whereas 3MA reverses Sit's antiapoptotic effects by inhibiting PI3K phosphorylation.

According to the concept of “angiosome” (Taylor and Minabe, 1992), the area of the donor flap is limited for pedicled flap and microsurgical flap transfer, so it is often limited in repairing some large wound. Both of these two types of flap transfer cannot be possible to cut more than two vascular territories flap, otherwise the distal part of the flap will be necrotic. Delayed procedures can promote the choke vessel's dilation, but it requires two times of operation, which is hard for patients to accept. In the present study, we found that Sit contributes to angiogenesis and choke vessel's dilation. It would be a useful method to improve the survival rate of pedicled flap and microsurgical flap transfer. What's more, oral administration is easy for patients to adopt. However, there is no evidence of clinical use of Sit to improve flap survival, which needs further experimental verification.

Our study had a few limitations which need to be addressed in future studies. First, despite having established that Sit-induced autophagy has detrimental effects on flap survival, an effective approach to inhibitory autophagy ought to be investigated. Second, it's not evident whether more factors are involved in the regulation of Sit therapy due to lack of vitro experiments. Third, this study only focused on effective concentration rather than concentration gradient to select an optimal dose. Nevertheless, this study presents Sit benefits in a perforator flap and lays a foundation for further research.

## Conclusions

Our study reveals that Sit has positive effects in promoting survival of multiterritory perforator flap, primarily by promoting angiogenesis, reducing apoptosis and oxidative stress accumulation. Additionally, effects of Sit-induced autophagy are detrimental for flap survival. Pro-angiogenesis and anti-oxidant stress are still enhanced to promote survival of perforator flap regardless of inhibition by Sit-induced autophagy which weakens anti-apoptotic effects.

## Data Availability Statement

The raw data supporting the conclusions of this article will be made available by the authors, without undue reservation, to any qualified researcher.

## Ethics Statement

The animal study was reviewed and approved by Animal Research Committee of Wenzhou Medical University.

## Author Contributions

ZC and CZ wrote the manuscript text. ZC, CZ, HM, ZH, and JLi prepared figures and collected samples. JLo, BL, and QT analyzed data. WG designed the experiment and revised the manuscript. All authors contributed to the article and approved the submitted version.

## Funding 

This work was supported by grants from China National Natural Science Foundation (81873942); Zhejiang Province Natural Science Foundation of China (LY15H060010).

## Conflict of Interest

The authors declare that the research was conducted in the absence of any commercial or financial relationships that could be construed as a potential conflict of interest.

## Supplementary Material

Figure S1 Chemical structure of Sitagliptin.

Figure S2 Sit and 3MA reduce flap swelling. **(A)** Digital photographs of the inner side of flap in the control, Sit, and Sit+3MA groups on the 7^th^ POD. **(B)** Histogram indicating the percentage of tissue water content in each group. *p < 0.05 vs control group; @p < 0.05 vs Sit group. Data are expressed as mean ± standard error, n = 6 per group.
