# Correction: Detrimental effect of sitagliptin induced autophagy on multiterritory perforator flap survival

**DOI:** 10.3389/fphar.2025.1677222

**Published:** 2026-01-23

**Authors:** Zhengtai Chen, Chenxi Zhang, Haiwei Ma, Zihuai Huang, Jiafeng Li, Junshen Lou, Baolong Li, Qi Tu, Weiyang Gao

**Affiliations:** 1 Department of Orthopaedics, The Second Affiliated Hospital and Yuying Children’s Hospital of Wenzhou Medical University, Wenzhou, China; 2 Department of Orthopaedics, Zhejiang Provincial Key Laboratory of Orthpaedics, Wenzhou, China; 3 Department of Second Clinical Medical, The Second Clinical Medical College of Wenzhou Medical University, Wenzhou, China; 4 Department of Neurosurgery, The First Affiliated Hospital of Wenzhou Medical University, Wenzhou, China; 5 Department of First Clinical Medical, The First Clinical Medical College of Wenzhou Medical University, Wenzhou, China

**Keywords:** multiterritory perforator flap, sitagliptin, autophagy, angiogenesis, apoptosis, oxidative stress, PI3K/Akt signaling pathway

There was a mistake in Figure 3 as published. The images of the control group and 3MA group in Figure 3C were mistakenly used, but their CTSD expression trends were consistent and did not affect the results. The corrected Figure 3 appears below.

**FIGURE 3:**
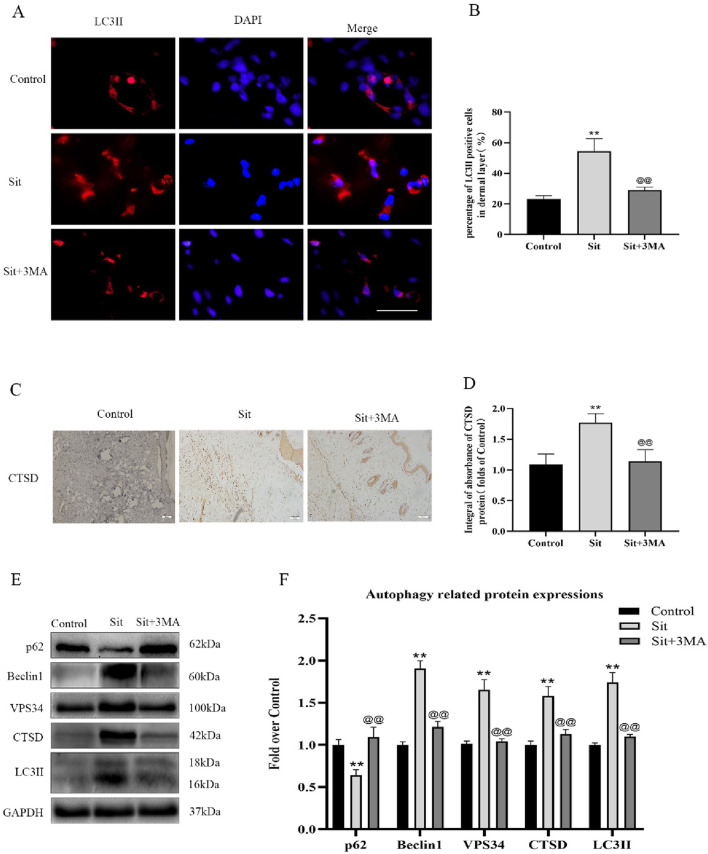
Sit induces autophagy in the perforator flap. **(A)** Autophagosomes (red) in cells in SCZ of flaps in the control, Sit, and Sit+3MA groups by immunofluorescence staining for LC3II (scale bar: 20 µm). **(B)** Histogram of fluorescence intensity of LC3II-positive cells in the dermal layer in each group. **(C)** IHC staining of CTSD expression in the dermis (original magnification: 200×; scale bar: 50 µm). **(D)** Histogram of CTSD level estimated by IHC. **(E)** Western blotting for Beclin1, VPS34, CTSD, SQSTM1/p62, and LC3II expressions in flap of the control, Sit, and Sit+3MA groups. All gels have been run under the same electrophoretic conditions and cropped blots are used here. **(F)** Histogram of autophagy related protein expressions of Beclin1, p62, CTSD, VPS34, and LC3II calculated as the folds of control. **p < 0.01, vs. control group; ^@@^p < 0.01, vs. Sit group. Data are presented as mean ± standard error, n = 6 per group.

There was a mistake in Figure 4 as published. The images in the Sit group of Figure 4E are not representative and need to be replaced with new images, but this will not affect the results. The corrected Figure 4 appears below.

**FIGURE 4:**
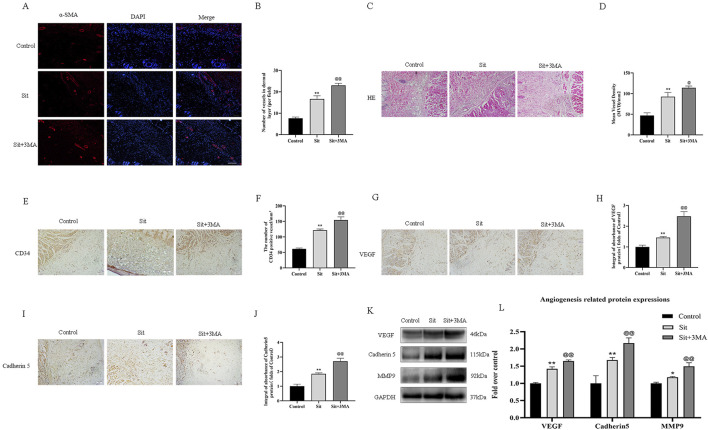
Effect of Sit-induced autophagy on angiogenesis in the perforator flap. **(A)** Microvessels (red) in SCZ of flaps in the control, Sit, and Sit+3MA groups were estimated by immunofluorescence staining for α-SMA in the dermal layer (scale bar: 20 µm). **(B)** Histograms representing percentages of α-SMA labeled microvessels in each group. **(C)** H&E staining exhibiting subcutaneous histology of the flap, showing microvessels in SCZ in the control, Sit, and Sit+3MA groups (original magnification ×200; scan bar, 50 μm). **(D)** Histogram indicating percentage of mean vessel density in each group. **(E)** IHC for CD34 positive vessels in the control, Sit, and Sit+3MA groups (original magnification ×200; scale bar, 50 µm). **(F)** Histogram of the percentage of CD34-positive vessel density in each group. **(G,I)** IHC for VEGF and Cadherin 5 expression in the flap in the control, Sit, and Sit+3MA groups (original magnification ×200; scale bar, 50 µm). **(H,J)** The optical density values of VEGF and Cadherin 5 in each group. **(K)** The expressions of MMP9, VEGF, and Cadherin 5 detected by western blotting in the control, Sit, and Sit+3MA groups. All gels have been run under the same experimental conditions and cropped blots are used here. **(L)** Histogram of the optical density values of MMP9, VEGF, and Cadherin 5 in each group. *p < 0.05 and **p < 0.01, vs. control group; ^@^p < 0.05 and ^@@^p < 0.01, vs. Sit group. Data are presented as mean ± standard error, n = 6 per group.

There was a mistake in Figure 5 as published. The images in the control group of Figure 5A are not representative and need to be replaced with new images, but this will not affect the results. The corrected Figure 5 appears below.

**FIGURE 5:**
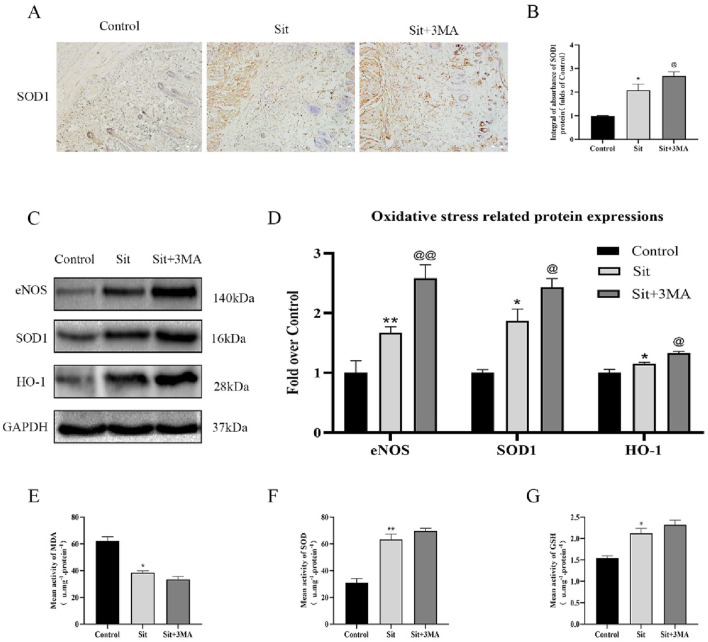
Effect of Sit-induced autophagy on oxidative stress in the perforator flap. **(A)** IHC for SOD1 expression in the perforator flap in the control, Sit, and Sit+3MA groups (original magnification ×200; scale bar, 50 µm). **(B)** Histogram of optical density values of SOD1 quantified and analyzed by IHC. **(C)** The expressions of SOD1, eNOS, and HO1 as revealed by western blotting in the control, Sit, and Sit+3MA groups. Gels have been run under the same experimental conditions and cropped blots are used here. **(D)** Histogram of oxidative stress related protein expressions of SOD1, eNOS, and HO1 in each group. **(E)** MDA level evaluated using modified thiobarbituric acid test. **(F)** Total SOD activity evaluated using xanthine oxidase method. **(G)** GSH level evaluated using modified 5,5′-dithiobis method. *p < 0.05 and **p < 0.01, vs. control group; ^@^p < 0.05 and ^@@^p < 0.01, vs. Sit group. Data are presented as mean ± standard error, n = 6 per group.

The original article has been updated.

