# Supplementary figures and images for: Detrimental Effect of Sitagliptin Induced Autophagy on Multiterritory Perforator Flap Survival
Source: Front Pharmacol. 2020 Jun 26;11:951. doi: 10.3389/fphar.2020.00951 (PMC7332881; doi:10.3389/fphar.2020.00951)

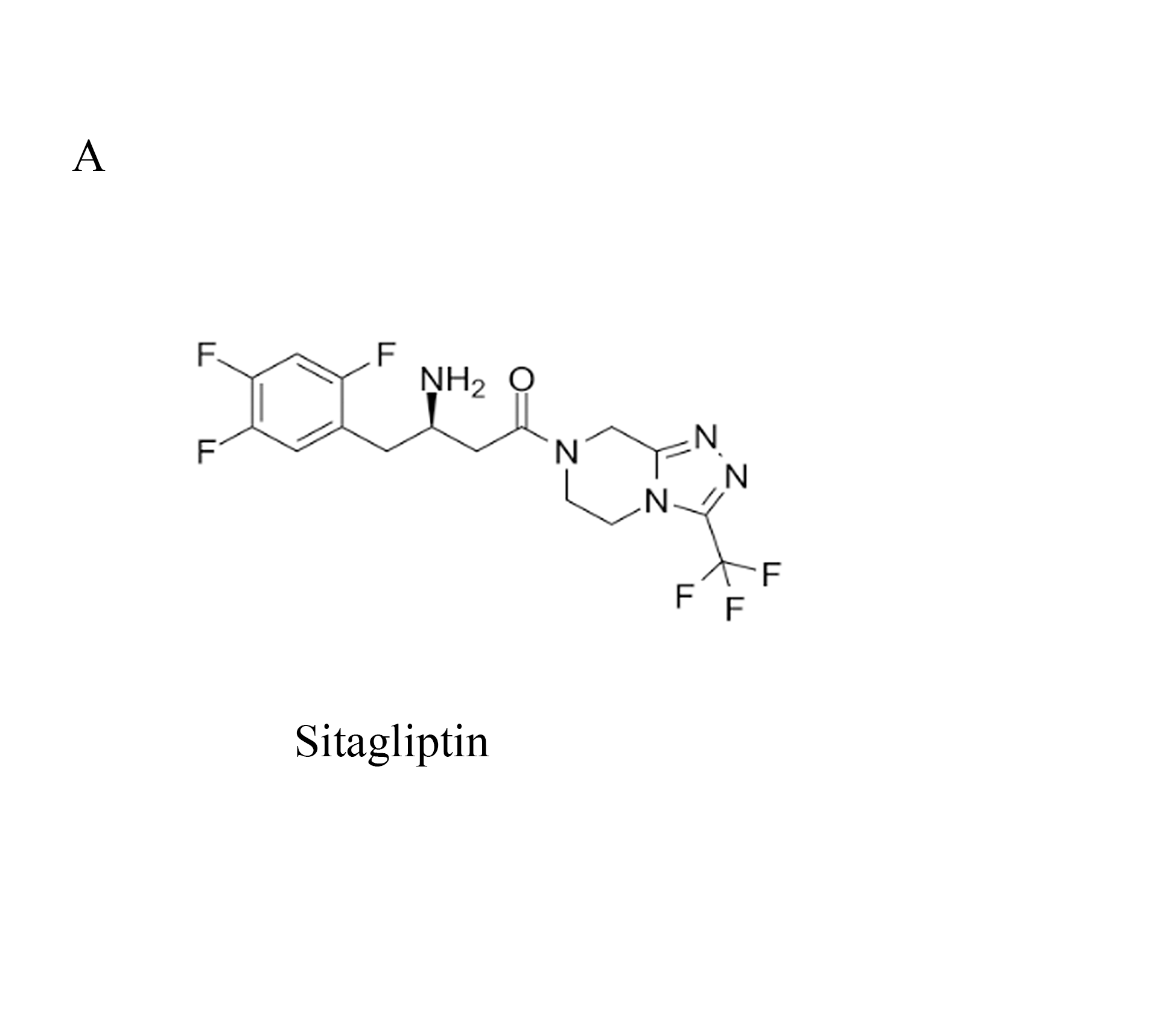

Supplement: Figure S1 — Chemical structure of Sitagliptin. [file Image_1.tif]

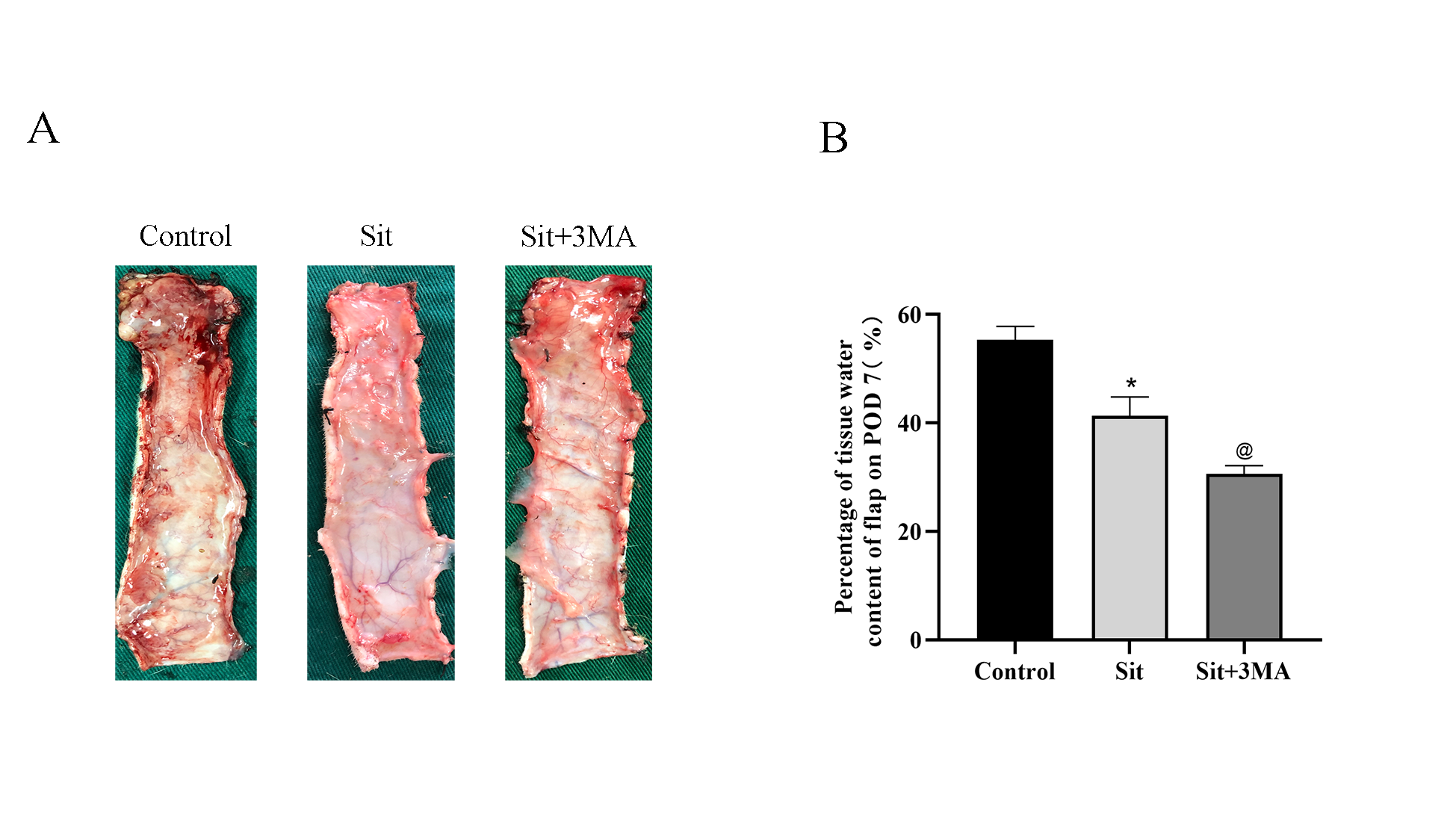

Supplement: Figure S2 — Sit and 3MA reduce flap swelling. (A) Digital photographs of the inner side of flap in the control, Sit, and Sit+3MA groups on the 7th POD. (B) Histogram indicating the percentage of tissue water content in each group. *p < 0.05 vs control group; @p < 0.05 vs Sit group. Data are expressed as mean ± standard error, n = 6 per group. [file Image_2.tif]
